# 1686. Bacteremia associated with intravascular catheter colonization with Gram-negative rods in children

**DOI:** 10.1093/ofid/ofad500.1519

**Published:** 2023-11-27

**Authors:** Hina Hisano, Kensuke Shoji, Toshihiro Matsui, Hiroki Kato, Kana Fukui, Motohiro Kano, Yuji Yamada, Yoshihiro Gocho, Akira Ishiguro

**Affiliations:** National Center for Child Health and Development, Setagaya-ku, Tokyo, Japan; National Center for Child Health and Development, Setagaya-ku, Tokyo, Japan; National Center for Child Health and Development, Setagaya-ku, Tokyo, Japan; National Center for Child Health and Development, Setagaya-ku, Tokyo, Japan; National Center for Child Health and Development, Setagaya-ku, Tokyo, Japan; National Center for Child Health and Development, Setagaya-ku, Tokyo, Japan; National Center for Child Health and Development, Setagaya-ku, Tokyo, Japan; National Center for Child Health and Development, Setagaya-ku, Tokyo, Japan; National Center for Child Health and Development, Setagaya-ku, Tokyo, Japan

## Abstract

**Background:**

Patients with *Staphylococcus aureus* colonization of an intravascular catheter, but without bacteremia, had a 14% chance of developing subsequent *S. aureus* bacteremia after removing the central venous catheter. However, information on the frequency and outcomes of subsequent bacteremia due to Gram-negative rods (GNRs) is limited, particularly in children.

**Methods:**

We conducted a single-center, retrospective, case-control study. Clinical information including age, sex, immune status, bacterial culture results from both catheter tips and the blood, and outcomes were extracted from electronic medical records. Patients were included if the central venous catheter tip culture was positive for GNRs, but the concurrent blood culture (within one day before or after catheter removal) was negative. Subsequent bacteremia was defined as bacteremia caused by the same organism(s) as the catheter tip culture within six months of catheter removal. Clinical characteristics were compared between patients with and without subsequent GNR bacteremia.

**Results:**

During the study period, 49 cases < 18 years of age that were positive for central venous catheter tip culture but negative for concurrent blood cultures were identified. Median (interquartile range) age was 0 (0-4.5) years and male sex was 44.9%. *Pseudomonas aeruginosa* was the most common isolate (n=18, 36.7%), followed by *Escherichia coli* (n=8, 16.3%) and *Klebsiella* spp. (n=8, 16.3%). Subsequent GNR bacteremia developed in 6 (12.2%) patients. The median (range) days between catheter removal and the occurrence of subsequent GNR bacteremia was 16.5 (3-35) days. The median age and the proportion of immunocompromised cases were higher in patients with subsequent bacteremia than in those without subsequent bacteremia (8 years *vs* 0 years, *P*=0.011 and 6 [100%] *vs* 21 [48.8%], *P*=0.027, respectively). The presence of antibiotic treatment at catheter removal was similar between these two groups (n=4 [66.7%] *vs* n=25 [58.1%], *P*=1.0).

**Conclusion:**

The frequency of subsequent GNR bacteremia in children was 12.2%. Immunocompromised patients may be at higher risk for developing subsequent GNR bacteremia.

**Disclosures:**

**Kensuke Shoji, MD, PhD**, AstraZeneca K.K.: Honoraria|Gilead Sciences, Inc.: Honoraria|KYORIN Pharmaceutical Co.,Ltd.: Honoraria|Meiji Seika Pharma Co., Ltd.: Honoraria|Nippon Becton Dickinson Company, Ltd.: Honoraria|Novartis Pharma Co., Ltd.: Honoraria|Viatrs, Inc: Honoraria

